# Obesity and the risk of gallbladder cancer: a meta-analysis

**DOI:** 10.1038/sj.bjc.6603703

**Published:** 2007-03-20

**Authors:** S C Larsson, A Wolk

**Affiliations:** 1 Division of Nutritional Epidemiology, The National Institute of Environmental Medicine, Karolinska Institutet, Box 210, SE-17177 Stockholm, Sweden

**Keywords:** body mass index, case–control studies, cohort studies, gallbladder cancer, meta-analysis, obesity

## Abstract

We performed a meta-analysis of studies of the association between excess body weight and risk of gallbladder cancer identified from MEDLINE and EMBASE databases from 1966 to February 2007 and the references of retrieved articles. A random-effects model was used to combine results from eight cohort studies and three case–control studies, with a total of 3288 cases. Compared with individuals of ‘normal weight’, the summary relative risk of gallbladder cancer for those who were overweight or obese was 1.15 (95% CI, 1.01–1.30) and 1.66 (95% CI, 1.47–1.88) respectively. The association with obesity was stronger for women (relative risk, 1.88; 95% CI, 1.66–2.13) than for men (relative risk, 1.35; 95% CI, 1.09–1.68). There was no statistically significant heterogeneity among the results of individual studies. This meta-analysis confirms the association between excess body weight and risk of gallbladder cancer.

Cancer of the gallbladder is a highly fatal malignancy, usually diagnosed at an advanced stage, with a 5-year survival rate of less than 10% (Misra *et al*, 2003). Although rare in most parts of Europe and in the United States, relatively high incidence and mortality rates are found in northern India, Chile, Pakistan, Korea and some eastern European countries (Randi *et al*, 2006). Gallbladder cancer is about two to five times more common in women than in men. Little is known about the aetiology of this neoplasm besides a strong link with gallstones (Randi *et al*, 2006). Obesity increases the risk for gallstones, and thus may be a risk factor of gallbladder cancer. Because the epidemiologic evidence on excess body weight and risk of gallbladder cancer has not yet been quantitatively summarised, we conducted a meta-analysis of cohort and case–control studies on this subject.

## METHODS

### Study selection

We conducted a computerised search of the MEDLINE and EMBASE databases for studies that were published from 1966 to February 2007, using the Medical Subject Headings or text words ‘body mass index’, ‘BMI’, ‘obesity’, or ‘weight’ combined with ‘gallbladder cancer’ or ‘gallbladder neoplasm’. In addition, we manually searched the reference lists of retrieved articles for additional relevant studies. No language restrictions were imposed.

Studies were included in the meta-analysis if they met the following criteria: (1) cohort or case–control study in which gallbladder cancer incidence or mortality was an outcome; (2) the exposure of interest was overweight and/or obesity defined by body mass index (BMI) (the weight in kilograms divided by the square of height in metres) and (3) estimates of relative risk (rate ratio, odds ratio, or standardised incidence ratio) with their corresponding 95% confidence intervals were reported. When there were multiple publications from the same study population, we included the one with the largest sample size.

We identified eight cohort studies (Møller *et al*, 1994; Wolk *et al*, 2001; Calle *et al*, 2003; Samanic *et al*, 2004, 2006; Engeland *et al*, 2005; Kuriyama *et al*, 2005; Oh *et al*, 2005) and four case–control studies (Zatonski *et al*, 1992, 1997; Strom *et al*, 1995; Serra *et al*, 2002) that reported results on overweight/obesity and risk of gallbladder cancer. One case–control study (Zatonski *et al*, 1992) was excluded because this study was included in a multicentre case–control study (Zatonski *et al*, 1997); the multicentre case–control study was included in this meta-analysis.

### Data extraction

For each study, the following data were extracted: first author's last name; publication year; country in which the study was performed; study design; source of controls (in case–control studies); sample size; method of assessment of weight and height (self-reported or measured); type of outcome (incidence or mortality); covariates for adjustments in the analysis and the risk estimates with corresponding 95% confidence intervals. From each study, we extracted the risk estimates that reflected the greatest degree of adjustment for potential confounders.

### Statistical analysis

Relative risk was used as the measure of the association between BMI and gallbladder cancer. Because gallbladder cancer is a rare disease, odds ratios and standardised incidence ratio provide a valid estimate of the relative risk; we report all results as relative risk for simplicity. The relative risks and corresponding standard errors (derived from the confidence intervals) from individual studies were logarithmically transformed to stabilise variances and normalise the distributions. We computed summary relative risk estimates for two categories of BMI as defined by the World Health Organisation (WHO) for adults: overweight (BMI 25–30 kg m^−2^) and obesity (BMI ⩾30 kg m^−2^) compared with ‘normal’ weight (BMI 18.5–24.9 kg m^−2^) as the reference category. Where nonstandard categories of BMI were used, we chose the category that was most similar to those defined by the WHO. Study-specific estimates were combined using the DerSimonian and Laird random-effects model, which incorporates both within- and between-study variation (DerSimonian and Laird, 1986).

Statistical heterogeneity among studies was evaluated with the *Q* and *I*^*2*^ statistics (Higgins and Thompson, 2002). For the *Q* statistic, statistical significance was set at *P*<0.1. A meta-regression analysis was performed to investigate whether the association between obesity and risk of gallbladder cancer differed by study design or sex. We conducted a sensitivity analysis, in which one study at a time was removed and the rest analysed to assess whether the results were markedly affected by a single study. We used funnel plots (i.e., plots of study results against precision) to assess publication bias, and tested its symmetry (Egger *et al*, 1997).

Results are presented graphically, whereby squares represent study-specific estimates and diamonds represent summary estimates. The area of each square is proportional to the inverse of the variance of the logarithm of the relative risk; 95% confidence intervals for individual studies are represented by horizontal lines and for the summary estimates by the width of the diamonds. All statistical analyses were performed with Stata, version 9.0 (StataCorp, College Station, TX, USA).

Population-attributable risk (PAR) for gallbladder cancer was estimated for individuals with excess body weight (BMI ⩾25 kg m^−2^) compared with those of normal weight (BMI <25 kg m^−2^). The PAR describes the theoretical proportion of cases that would be prevented if all individuals were moved into the exposure level associated with the lowest risk for that factor. The PAR (PAR%) was calculated as PAR%=(*p* × (*RR*−1)/(*p* × (*RR*−1)+1)) × 100%, where *p* represents the prevalence in the population and *RR* the relative risk. Prevalence data were obtained from the National Health and Nutrition Examination Survey that assessed the prevalence of overweight and obesity in a representative sample of the US population (Ogden *et al*, 2006). In that survey, 39.7% of the men were overweight and 31.1% were obese. Among women, 28.6% were overweight and 33.2% were obese. PARs were calculated for the overweight and obese categories using the obtained summary relative risk estimates, and then summarised across the two categories for men and women separately.

## RESULTS

The eight cohort studies (Møller *et al*, 1994; Wolk *et al*, 2001; Calle *et al*, 2003; Samanic *et al*, 2004, 2006; Engeland *et al*, 2005; Kuriyama *et al*, 2005; Oh *et al*, 2005) and three case–control studies (Strom *et al*, 1995; Zatonski *et al*, 1997; Serra *et al*, 2002) that were included in the meta-analysis involved a total of 3288 cases. Main characteristics of the studies are shown in Table 1. Weight and height were measured in three studies (Engeland *et al*, 2005; Oh *et al*, 2005; Samanic *et al*, 2006) and self-reported in five studies (Strom *et al*, 1995; Zatonski *et al*, 1997; Serra *et al*, 2002; Calle *et al*, 2003; Kuriyama *et al*, 2005); in three studies, obesity was defined by a discharge diagnosis of obesity (Møller *et al*, 1994; Wolk *et al*, 2001; Samanic *et al*, 2004). Among case–control studies, one used population-based controls (Zatonski *et al*, 1997) and two used hospital-based controls (Strom *et al*, 1995; Serra *et al*, 2002).

**Figure 1:**
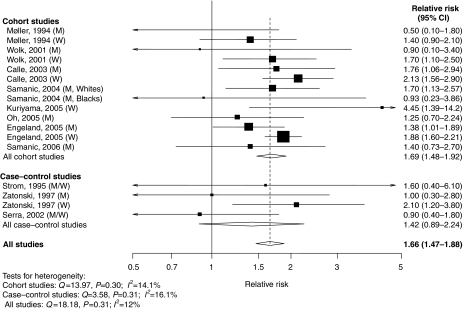
Relative risks of gallbladder cancer associated with obesity. Relative risk estimates are for comparison of individuals in the ‘obese’ category compared to those with ‘normal weight’. M=men; W=women.

Relative risk estimates of gallbladder cancer for ‘obesity’ compared with ‘normal weight’ in individual studies (separately for men and women wherever this information was available) and all studies combined are shown in Figure 1. Of the 18 relative risk estimates from the 11 studies, 14 were above one, of which eight were statistically significant. Meta-analysis of all studies found that compared to individuals with ‘normal weight’, those who were ‘obese’ had a 66% increased risk with no statistically significant heterogeneity among individual studies (Figure 1). In a sensitivity analysis, no single study appreciably influenced the summary relative risk, which ranged from 1.59 to 1.73 when omitting individual studies one at a time. The relation with obesity did not differ – statistically – significantly by study design (*P*=0.56) (Figure 1). However, the summary estimate was higher (*P*=0.02) for women (RR, 1.88; 1.66–2.13) than for men (RR, 1.35; 95% CI, 1.09–1.68). Summary relative risks were 1.82 (95% CI, 1.37–2.42) for studies based on self-report, 1.60 (95% CI, 1.29–1.98) for those with weight and height measurements and 1.51 (95% CI, 1.20–1.91) for those based on a discharge diagnosis of obesity. There was some indication of publication bias on the funnel plot (data not shown), suggesting the relative absence of small studies with null results; the Egger's test yielded a *P*-value of 0.05.

Eight studies presented relative risk estimates for ‘overweight’ compared with ‘normal weight’ (Strom *et al*, 1995; Zatonski *et al*, 1997; Serra *et al*, 2002; Calle *et al*, 2003; Engeland *et al*, 2005; Kuriyama *et al*, 2005; Oh *et al*, 2005; Samanic *et al*, 2006). Meta-analysis of these studies found those in the overweight category had a statistically significant 15% increased risk (RR, 1.15; 95% CI, 1.01–1.30) compared to those of normal weight, with no statistically significant heterogeneity among studies (*Q*=14.93; *P*=0.19; *I*^2^=26.3). The summary relative risk did not differ – statistically – significantly by study design (*P*=0.86), but was higher (*P*=0.09) for women (RR, 1.28; 95% CI, 1.04–1.57) than men (RR, 1.05; 95% CI, 0.92–1.19). We found no evidence of publication bias on the funnel plot (data not shown) or by Egger's test (*P*=0.87).

The PAR for excess body weight was calculated using the estimates of prevalence in the United States (Ogden *et al*, 2006) and the obtained summary relative risks for overweight (1.05 for men and 1.28 for women) and obesity (1.35 for men and 1.88 for women). We estimated that 12% of the gallbladder cancer cases among men and 30% among women could be attributable to excess body weight (BMI ⩾25 kg m^−2^).

## DISCUSSION

This meta-analysis of published studies indicates that the risk of gallbladder cancer increases with increasing BMI. Summary results showed that gallbladder cancer risk was 15 and 66% higher among those who were overweight and obese, respectively, as compared with those of normal weight. The association between obesity and gallbladder cancer risk was stronger in women than in men.

Several potential limitations should be considered when interpreting the results from this meta-analysis. First, because of the observational design of the included studies, we cannot rule out the possibility that the observed association between excess body weight and risk is owing to confounding from other risk factors. Second, five studies (Strom *et al*, 1995; Zatonski *et al*, 1997; Serra *et al*, 2002; Calle *et al*, 2003; Kuriyama *et al*, 2005) in this meta-analysis relied on self-reported weight and height and it is possible that weight has been under-reported, particularly by overweight or obese individuals (IARC, 2002). The summary relative risk estimate was higher for studies that relied on self-reported weight and height. In case–control studies, weight loss before the cancer diagnosis would attenuate the relative risk estimates. The summary relative risk estimate was slightly lower for case–control studies than for cohort studies. Findings from case–control studies could also be biased if individuals in the control group were more health conscious and thus less likely to be overweight or obese than the cases. Finally, there was some suggestion of publication bias. The presence of publication bias could have led to an overestimation of the true association between obesity and risk of gallbladder cancer.

Potential biological mechanisms for the association include increased concentrations of endogenous hormones (estrogens, insulin, and insulin-like growth factor-I) in overweight and obese individuals. Obesity may increase risk indirectly by increasing the risk of gallstones, which, in turn, increase the risk for gallbladder cancer (Randi *et al*, 2006). The reason for the stronger association observed with obesity in women than in men is unclear. The three studies that found that obesity was associated with an increased risk in men only were all based on a small number of male cases in the obese category (Møller *et al*, 1994; Zatonski *et al*, 1997; Wolk *et al*, 2001), so the stronger association observed in women may be due to chance.

In summary, this meta-analysis indicates that excess body weight is a risk factor for gallbladder cancer, suggesting that it may in part be prevented by maintaining a healthy body weight.

## Figures and Tables

**Table 1:** Characteristics of the 11 studies included in the meta-analysis

					**Relative risk**^a^ **(95% CI)**		
**Study (country)**	**Study design**	**Cases (men/women)**	**Controls or cohort size (men/women)**	**BMI (kg m^–2^) categories**	**Men**	**Women**	**Adjustments**
Møller *et al*, 1994 (Denmark)	Cohort	2/26	Cohort	Non-obese	1.0 (reference)	1.0 (reference)	Age
			14 531/29 434	Obese	0.5 (0.1–1.8)	1.4 (0.9–2.1)	
Strom *et al*, 1995 (Mexico)	C–C	65	110		Men and women		Age, sex
				<24.0	1.0 (reference)		
				24.0–25.9	1.5 (0.5–4.6)		
				26.0–28.0	2.2 (0.7–8.4)		
				>28.0	1.6 (0.4–6.1)		
Zatonski *et al*, 1997 (Multicenter^b^)	C–C	44/145	798/681	Quartile 1	1.0 (reference)	1.0 (reference)	Age, center, education, alcohol, smoking, response status
				Quartile 2	1.0 (0.3–3.0)	1.7 (0.9–3.1)	
				Quartile 3	0.7 (0.3–2.0)	1.5 (0.8–3.0)	
				Quartile 4	1.0 (0.3–2.8)	2.1 (1.2–3.8)	
Wolk *et al*, 2001 (Sweden)	Cohort	2/29	Cohort	Non-obese	1.0 (reference)	1.0 (reference)	Age, calendar year
			8165/19 964	Obese	0.9 (0.1–3.4)	1.7 (1.1–2.5)	
Serra *et al*, 2002 (Chile)	C–C	114	114		Men and women		Age, sex
				<25.0	1.0 (reference)		
				25.0–29.9	0.8 (0.4–1.4)		
				⩾30.0	0.9 (0.4–1.8)		
Calle *et al*, 2003 (United States)	Cohort	180/304	Cohort	18.5–24.9	1.00 (reference)	1.00 (reference)	Age, race, marital status, education,
			404 576/495 477	25.0–29.9	1.34 (0.97–1.84)	1.12 (0.86–1.47)	smoking, physical activity, aspirin use, estrogen-replacement therapy (women), alcohol, dietary factors
				⩾30.0	1.76 (1.06–2.94)	2.13 (1.56–2.90)	
Samanic *et al*, 2004 (United States)	Cohort	291 whites/	Cohort		White men	Black men	Age, calendar year
		47 blacks	366 8486 white	Non-obese	1.00 (reference)	1.00 (reference)	
			men/832 214 black men	Obese	1.70 (1.13–2.57)	0.93 (0.23–3.86)	
Kuriyama *et al*, 2005 (Japan)	Cohort	9/24	Cohort	18.5–24.9	1.00 (reference)	1.00 (reference)	Age, smoking, type of health
			12 485/15 054	25.0–27.4	0.46 (0.05–3.93)^c^	0.83 (0.23–2.98)	insurance, intakes of alcohol, meat,
				27.5–29.9		3.43 (1.19–9.94)	fish, fruits, vegetables, bean-paste
				⩾30.0		4.45 (1.39–14.2)	soup^d^
Oh *et al*, 2005 (Korea)	Cohort	182	Cohort	18.5–22.9	1.00 (reference)		Age, area of residence, smoking,
			781 283/–	23.0–24.9	1.55 (1.10–2.20)		exercise, alcohol
				25.0–26.9	1.15 (0.74–1.80)		
				⩾27.0	1.25 (0.70–2.24)		
Engeland *et al*, 2006 (Norway)	Cohort	628/1087	Cohort	18.5–24.9	1.00 (reference)	1.00 (reference)	Age, birth cohort
			962 901/1037 077	25.0–29.9	1.00 (0.84–1.17)	1.27 (1.10–1.47)	
				⩾30.0	1.38 (1.01–1.89)	1.88 (1.60–2.21)	
Samanic *et al*, 2006 (Sweden)	Cohort	109	Cohort	18.5–24.9	1.00 (reference)		Age, smoking
			362 552/–	25.0–29.9	0.93 (0.62–1.39)		
				⩾30.0	1.40 (0.73–2.70)		

BMI=body mass index; C–C=case–control study; CI=confidence interval.

a Relative risks are rate ratios, odds ratios, or standardised incidence ratios.

b Multicentre case–control study conducted in five centres located in Australia (Adelaide), Canada (Montreal and Toronto), The Netherlands (Utrecht), and Poland (Opole).

c The highest BMI category for men was 25.0–27.4 kg m^–2^; there was only one case in this category.

d Odds ratios for women were further controlled for age at menarche, age at end of first pregnancy, and menopausal status.
